# Bilateral functional hippocampectomy following recurrent bacterial meningitis: a case report

**DOI:** 10.1186/s13256-026-05993-1

**Published:** 2026-04-07

**Authors:** Marija Djukic, Jörg Larsen, Hilmar Prange, Veit Rohde, Roland Nau

**Affiliations:** 1 Department of Geriatrics, Evangelisches Krankenhaus Göttingen-Weende, An der Lutter 24, 37075 Göttingen, Germany; 2 Department of Radiology, Evangelisches Krankenhaus Göttingen-Weende, An der Lutter 24, 37075 Göttingen, Germany; 3 Department of Neurology, University Medical Center, Göttingen, Germany; 4 Department of Neuropathology, University Medical Center, Göttingen, Germany; 5 Department of Radiology, University Medical Center, Göttingen, Germany; 6 Department of Neurosurgery, University Medical Center, Göttingen, Germany

**Keywords:** Meningitis, *Listeria monocytogenes*, *Acinetobacter baumannii*, *Staphylococcus epidermidis*, Hippocampus atrophy, Magnetic resonance imaging, Learning deficit

## Abstract

**Background:**

The hippocampal formation is indispensable to convert short-term into long-term memories. It is a brain region particularly vulnerable to a variety of noxious stimuli. Severe hippocampal injury with complete inability to convert short-term into long-term memories is very rare in bacterial meningitis.

**Case presentation:**

At the age of 62 years, a white German female teacher acquired *Listeria monocytogenes* meningitis after the consumption of goat cheese manufactured from raw milk. Malabsorptive hydrocephalus required placement of a ventriculoperitoneal shunt. The further course became complicated by two shunt infections. Severe bilateral hippocampal atrophy developed. In spite of restoration of CSF drainage via a replacement shunt, the patient lost the capacity to form new memories. Conversely, her personality and the ability to work with previously stored information remained intact for many years.

**Conclusions:**

Protection of the hippocampal formation and preservation of the capacity to form new memories is a major goal in the treatment of bacterial meningitis. Further improvement of outcome in bacterial meningitis will depend on approaches that effectively protect the hippocampal formation.

## Background

The hippocampal formation is indispensable to convert short-term into long-term memories. Bilateral hippocampectomy resulted in a „very grave recent memory loss, so severe as to prevent the patient from remembering the locations of the rooms in which he lives, the names of his close associates, or even the way to toilet and urinal [1]. Hippocampal atrophy is a prominent feature of Alzheimer's disease, and volumetry of the hippocampal formation is used for its diagnosis and prediction. The volume of the hippocampus correlates with the cognitive decline in Alzheimer`s disease [2]. Acute hippocampal damage occurs as a consequence of many diseases, including hypoxia, hypoglycaemia, sepsis and bacterial meningitis [3–7]. Here, we present the case of an almost complete loss of the ability to convert short-term into long-term memories after bacterial meningitis.

## Case presentation

### History

During a vacation on the Canary Islands, a 62-year-old white German female previously healthy teacher developed headache, nausea and vomiting. Lumbar puncture after hospital admission yielded CSF leukocytes of 260/μl (60% lympho- and monocytes, 40% granulocytes), a CSF total protein of 1460 mg/l, and growth of *Listeria monocytogenes* (Table 1). Blood cultures also grew *L. monocytogenes*, the blood leukocyte count was 12,300/μl. The probable source of infection was the consumption of goat cheese manufactured from raw milk. The patient was treated with ampicillin plus gentamicin intravenously (i.v.). Three days after admission, she was in a comatose state, requiring intubation and artificial ventilation. Cranial computed tomography revealed cerebral edema and hydrocephalus. For brain edema, the patient was treated with corticosteroids > 3 weeks and mannitol. For prolonged coma, she required tracheotomy. For hydrocephalus, 1 week after admission an external ventriculostomy was implanted. One week later, the comatose, spontaneously breathing patient was transferred to our institution. Upon admission, the left pupil was mydriatic, the light reaction was delayed on both eyes, and the corneal reflex was weak on both sides. No spontaneous movements were present, but the patient reacted to painful stimuli by slight grimacing and withdrawing her extremities. The tendon reflexes were brisk. Babinski`s sign was absent on both sides.

**Table 1:** Timeline of meningitis caused by *Listeria monocytogenes* and ventriculitis caused by *Acinetobacter baumannii* and *Staphylococcus epidermidis*

Clinical event	Patient’s age (years)	Intervention	Result
*L. monocytogenes* meningitis	62	Ampicillin 12 g/d i.v. for 50 days, gentamicin 320 mg/d i.v. for 14 days	Coma, hydrocephalus, external ventricular drain
15 days after onset of *L. monocytogenes* meningitis	62	1st MRI	Moderate hippocampal atrophy
*A. baumannii ventriculitis*, 20 days after onset of *L. monocytogenes* meningitis	62	Moxifloxacin, imipenem, rifampicin i.v., intrathecal gentamicin and amikacin for a total of 5 weeks	Awake, disoriented to time, biventriculoperitoneal shunt
*S. epidermidis* ventriculitis	66	Vancomycin 20 mg/d intraventricularly for 3 days, cefazolin 3 × 2 g for 12 days	Awake, disoriented to time, ventriculoperitoneal shunt
12 days after onset of *S. epidermidis* meningitis	66	2nd MRI	Severe hippocampal atrophy
Severe learning disability, dizziness, anxiety, able to live at home	62–82	Physiotherapy, support by husband, family and friends	Able to live in her house
Frailty	82–84	Physiotherapy	Nursing home

i.v., intravenous; *A., Acinetobacter; L., Listeria; S., Staphylococcus;* MRI, magnetic resonance imaging

Five days later, the patient developed a ventricular drain infection (isolated pathogen Acinetobacter baumannii), which was treated antibiotically and required an exchange of the external ventriculostomy. Ampicillin 12 g/d was administered i.v. for a total of 50 days, and gentamicin was co-administered i.v. for the first 14 days. The subsequent ventricular drain infection required intravenous treatment with moxifloxacin, imipenem, rifampicin and intrathecal gentamicin and amikacin for a total of 5 weeks. The external ventriculostomy was changed twice, and after the CSF had become sterile, 2 months after disease onset a biventriculoperitoneal shunt was implanted.

One week later, upon discharge into a rehabilitation hospital, the patient was oriented concerning her person, but disoriented with respect to time. After 3 months of rehabilitation, the patient was discharged home with an Activity of Daily Living (Barthel) Index of 30 (out of 100), lacking in drive despite treatment with citalopram and still temporally disoriented.

Four years after the first ventriculoperitoneal shunt, the patient suffered from ventriculitis caused by *Staphylococcus epidermidis* after an accidental puncture of the shunt during a skin biopsy, which again required shunt removal (Table 1). The patient was treated with cefazolin i.v. for 12 days and received vancomycin intraventricularly for 3 days. A ventriculocisternostomy was performed, but this procedure did not suffice in treating her hydrocephalus. Therefore, another ventriculoperitoneal shunt was implanted into the frontal horn of the right lateral ventricle.

During the whole course of the disease, no epileptic seizures were noted. Therefore, the patient did not receive antiepileptic drug therapy. Because of the lack of clinical suspicion, testing for paraneoplastic or autoimmune encephalitis was not performed.

### Magnetic resonance imaging (MRI)

**Fig. 1:**
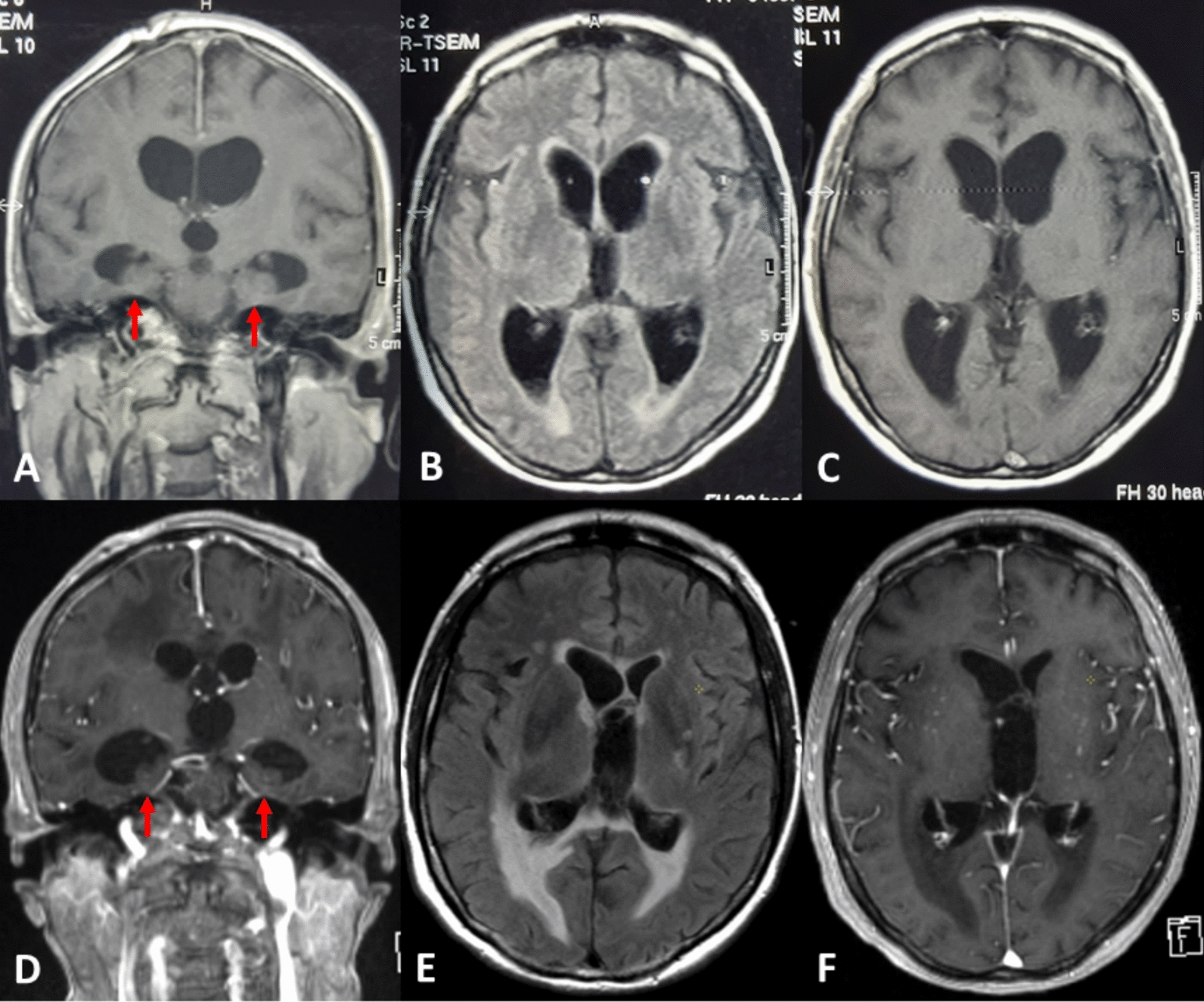
Cranial magnetic resonance (MR) images with coronal sections at the level of the hippocampal formation (**A**, **D**) and transverse-axial sections at the level of the third ventricle (**B**, **C** and **E**, **F**). Fifteen days after admission for *Listeria monocytogenes* meningoencephalitis (top row), the images show symmetric moderate ventriculomegaly with initial hippocampal volume loss (arrows) (T1-weighting, **A**), small high signal intensity seams in FLAIR weighting in the border areas of the vascular territories (**B**), and in a contrast medium enhanced T1-weighted sections a lack of ependymal enhancement in the ventricular system (**C**). Four years later (bottom row), 12 days after admission for *S. epidermidis* ventriculitis, the same sections show disproportionate dilatation of the temporal horns due to hippocampal volume loss (arrows) (contrast-enhanced T1-weighting, **D**). In addition, high signal intensity in the FLAIR weighting now outlines the entire margins of the ventricular system (**E**). Again, there is a lack of any pathological contrast medium enhancement of the ventricular ependyma (**F**). Please note the septal distortion at the opening of the lateral ventricles into the third ventricle at the later state, consistent with ventricular scarring due to repeated ventriculitis (**E**, **F**)

MRI [T1-weighted images ± gadolinium contrast enhancement, T2-weighted images, fluid attenuated inversion recovery (FLAIR) sequences] was performed 15 days after hospital admission for meningitis caused by *L. monocytogenes* and 4 years later,12 days after admission for *S. epidermidis* ventriculitis (Fig. 1). At both timepoints, coronal images permitted the assessment of the hippocampal size.

### Neuropsychology

The neuropsychological deficits of the patient were clearly described by her husband 4.5 years after *L. monocytogenes* meningitis:

The lack in drive persisted despite medical treatment with citalopram and sertralin. The loss of the capacity to form new memories persisted. The patient was able to read fluently, but unable to memorize what she had read. As a retired teacher, she was able to correct even long texts for spelling and grammar mistakes. She solved crosswords and recalled events and the content of books which she had read prior to the *L. monocytogenes* meningitis without problems. The patient enjoyed travelling to known places and visiting close relatives and friends, but was disoriented and fearful in unknown environments.

The second ventricular shunt infection 4 years after *L. monocytogenes* meningitis aggravated these deficits. The patient developed fatigue and started sleeping 14–15 h daily (12 h at night, 2–3 h at noon).

Clock-drawing tests performed 11 and 13 years after *L. monocytogenes* meningitis were normal. Mini Mental Status Examination (MMSE) 18 years after *L. monocytogenes* meningitis revealed temporal and spatial disorientation: the patient was unable to report the month, the day of the week, the date, the town, the name of the hospital, and the floor level of the hospital, where the consultation took place. She repeated the names of three items without difficulties, but was unable to recall these items a few minutes later and to repeat a longer sentence. Conversely, her language was completely preserved, she named items correctly without delay, performed complex demands, read and wrote sentences and drew two interlaced pentagons without problems. In total, she scored 20 of 30 points.

The preservation of language, executive function, visuospatial skills, and procedural memory argued against diffuse cortical dementia. The normal clock-drawing tests and the preserved complex task execution supported selective hippocampal dysfunction.

The geriatric depression scale was 0, indicating no mood abnormality.

### Repeated neurological examinations

During repeated control examinations at 6 month intervals, the patient frequently complained of dizziness and a blurred vision. Mild dysphagia without cranial nerve paralyses was noted. Nuchal rigidity and papilledema were absent. The ventriculoperitoneal shunt function was normal. The Romberg test with open and closed eyes was unstable, but possible for > 10 s. Maximum time of standing on one leg was 5 s. No nystagmus and no abnormalities of eyeball movements were noted. The finger perimetry was normal. Paralyses of the extremities, spasticity or rigidity were absent. The triceps surae reflex was weak, the other tendon reflexes were normal, Babinski`s sign was absent. The recognition of vibration at both lower extremities was mildly reduced, other sensory abnormalities were absent. The patient had no ataxia. Free gait was fearful, but the patient was able to walk without falls.

### Social conditions

Despite her severe deficit to generate memories, the patient was able to live in her house for 20 years, first supported by her husband, after his death by her family, friends and professional staff (Table 1). Because of this strong support, her subjective quality of life was high in spite of the persistent inability to form memories. At the age of 82, she moved into a nursing home. In the following years, her motor and cognitive status slowly deteriorated, and she became bedridden for most of the day. 22 years after surviving *L. monocytogenes* meningoencephalitis she died.

## Conclusions and discussion

The hippocampus, indispensable for the formation of memories, possesses a particular vulnerability to several forms of acute injury, including infection. One reason for this vulnerability is the blood supply via branches of the posterior cerebral artery receiving its blood mainly from the basilar artery and via the anterior choroidal artery, a branch of the internal carotid artery 2–5 mm prior to the bifurcation of the anterior cerebral and middle cerebral arteries. The long course of the supplying arteries and the relatively small number of capillary anastomoses are reasons for the inadequate ability of the hippocampus to cope with hypoperfusion. Another reason for the vulnerability of the hippocampal formation is the presence of densely packed excitatory neurons in the pyramidal cell layer of the hippocampus und the granular cell layer of the dentate gyrus predisposing these regions to excitotoxic injury as a consequence of glutamate release after repetitive neuronal depolarization. Autopsy [8] and 7 Tesla MRI studies [9] demonstrated a high interindividual variation of the hippocampal arterial supply explaining differences in individual hippocampal susceptibility to noxious stimuli. In the present case, hippocampal injury probably was a consequence of hypoperfusion in conjunction with toxic and pro-inflammatory bacterial products causing microglial activation and leukocyte migration and aggravated by corticosteroid administration [5, 10–13]. To our knowledge, the patient never experienced systemic hypoxia or epileptic seizures. Since the neurotoxicity of the antibiotics administered to the present patient usually is mediated by epileptic seizures, antibiotic-induced neurotoxicity as the cause of hippocampal injury in the present case is very unlikely.

This case report documents an extreme damage to both hippocampi, initiated by *L. monocytogenes* meningitis and aggravated by *A. baumannii* and *S. epidermidis* ventriculitis. Because of the severity of the initial *L. monocytogenes* meningoencephalitis (prolonged coma, strong meningeal inflammation, development of hydrocephalus, slow recovery, early hippocampal atrophy after 15 days), we consider the neurolisteriosis the strongest damaging event. Subsequent ventriculitis by *A. baumannii* and *S. epidermidis* contributed to the worsening of hippocampal function, but by itself would not have caused the severe cognitive symptoms.

The strengths of this report are the thorough description of the neuropsychological deficits by the family and the long observation period. The limitation is the relatively scarce use of established neuropsychological tests.

Corticosteroids probably are not the ideal agents to protect the hippocampus in critical illness [10, 11]. In experimental adult and infant bacterial meningitis [12, 13], adjunctive dexamethasone increased neuronal apoptosis in the dentate gyrus of the hippocampal formation. In adults with community-acquired bacterial meningitis in high-income countries, a detrimental effect of dexamethasone on hippocampal function, however, has never been proven. In adults with *S. pneumoniae* meningitis, the beneficial effect on brain oedema, inflammation and the associated improvement of cerebral perfusion and CSF flow appears to outweigh the detrimental action of corticosteroids on the hippocampus. Here, adjunctive dexamethasone reduced mortality, hearing loss and overall neurological sequelae [14]. This positive effect was absent in adult *Haemophilus influenzae* and *Neisseria meningitidis* meningitis. Whether adjunctive dexamethasone is protective or detrimental in *L. monocytogenes* meningitis is still a matter of debate [15, 16]. Adjunctive dexamethasone is not effective in nosocomial bacterial meningitis and under the conditions of developing countries.

In conclusion, dexamethasone or another corticosteroid does not appear to be the ideal adjunctive therapy for the protection of the hippocampal formation in bacterial meningitis. Approaches, which may protect the hippocampus in bacterial meningitis are the use of non-bacteriolytic bactericidal antibiotics [17–19], possibly in conjunction with more specific anti-inflammatory agents, such as matrix metalloproteinase inhibitors or radical scavengers [20]. We are confident that improved treatment protocols for bacterial meningitis designed to equally protect the hippocampus and the neocortex will help to avoid deleterious long-term sequelae as in the present case.

## Data Availability

Anonymized patient data will be provided by the corresponding author upon reasonable request, unless the anonymity of the patient may be endangered.

## Abbreviations

*A*.: *Acinetobacter*
CSF: Cerebrospinal fluid
FLAIR: Fluid attenuated inversion recovery
i.v.: Intravenous
*L*.: *Listeria*
MMSE: Mini Mental Status Examination
MR: Magnetic resonance
MRI: Magnetic resonance imaging
*S**.*: *Staphylococcus*

## References

[1] Scoville WB. The limbic lobe in man. J Neurosurg. 1954;11(1):64–6. 10.3171/jns.1954.11.1.0064. 13131095 10.3171/jns.1954.11.1.0064

[2] Yavuz BB, Ariogul S, Cankurtaran M, Oguz KK, Halil M, Dagli N, Cankurtaran ES. Hippocampal atrophy correlates with the severity of cognitive decline. Int Psychogeriatr. 2007;19(4):767–77. 10.1017/S1041610206004303. 17005070 10.1017/S1041610206004303

[3] Auer RN. Hypoglycemic brain damage. Metab Brain Dis. 2004;19(3–4):169–75. 15554413 10.1023/b:mebr.0000043967.78763.5b

[4] Schmidt-Kastner R. Genomic approach to selective vulnerability of the hippocampus in brain ischemia-hypoxia. Neuroscience. 2015;309:259–79. 10.1016/j.neuroscience.2015.08.034. 26383255 10.1016/j.neuroscience.2015.08.034

[5] Nau R, Soto A, Brück W. Apoptosis of neurons in the dentate gyrus in humans suffering from bacterial meningitis. J Neuropathol Exp Neurol. 1999;58(3):265–74. 10.1097/00005072-199903000-00006. 10197818 10.1097/00005072-199903000-00006

[6] Mazeraud A, Pascal Q, Verdonk F, Heming N, Chrétien F, Sharshar T. Neuroanatomy and physiology of brain dysfunction in sepsis. Clin Chest Med. 2016;37(2):333–45. 10.1016/j.ccm.2016.01.013. 27229649 10.1016/j.ccm.2016.01.013

[7] Tauber SC, Eiffert H, Brück W, Nau R. Septic encephalopathy and septic encephalitis. Expert Rev Anti Infect Ther. 2017;15(2):121–32. 10.1080/14787210.2017. 27885885 10.1080/14787210.2017.1265448

[8] Erdem A, Yaşargil G, Roth P. Microsurgical anatomy of the hippocampal arteries. J Neurosurg. 1993;79(2):256–65. 10.3171/jns.1993.79.2.0256. 8331410 10.3171/jns.1993.79.2.0256

[9] Spallazzi M, Dobisch L, Becke A, Berron D, Stucht D, Oeltze-Jafra S, Caffarra P, Speck O, Düzel E. Hippocampal vascularization patterns: a high-resolution 7 Tesla time-of-flight magnetic resonance angiography study. Neuroimage Clin. 2019;21: 101609. 10.1016/j.nicl.2018.11.019. 30581106 10.1016/j.nicl.2018.11.019 PMC6413539

[10] Sousa N, Almeida OF. Corticosteroids: sculptors of the hippocampal formation. Rev Neurosci. 2002;13(1):59–84. 10.1515/revneuro.2002.13.1.5. 12013026 10.1515/revneuro.2002.13.1.59

[11] Hill AR, Spencer-Segal JL. Glucocorticoids and the Brain after Critical Illness. Endocrinology. 2021;162(3): bqaa242. 10.1210/endocr/bqaa242. 33508121 10.1210/endocr/bqaa242 PMC7846201

[12] Zysk G, Brück W, Gerber J, Brück Y, Prange HW, Nau R. Anti-inflammatory treatment influences neuronal apoptotic cell death in the dentate gyrus in experimental pneumococcal meningitis. J Neuropathol Exp Neurol. 1996;55(6):722–8. 10.1097/00005072-199606000-00006. 8642398 10.1097/00005072-199606000-00006

[13] Leib SL, Heimgartner C, Bifrare Y-D, Loeffler JM, Täuber MG. Dexamethasone aggravates hippocampal apoptosis and learning deficiency in pneumococcal meningitis in infant rats. Pediatr Res. 2003;54:353–7. 10.1203/01.PDR.0000079185.67878.72. 12788989 10.1203/01.PDR.0000079185.67878.72

[14] Brouwer MC, McIntyre P, Prasad K, van de Beek D. Corticosteroids for acute bacterial meningitis. Cochrane Database Syst Rev. 2015;9:CD004405. 10.1002/14651858.CD004405.pub5 PMC6491272 26362566

[15] Charlier C, Perrodeau É, Leclercq A, *et al*. Clinical features and prognostic factors of listeriosis: the MONALISA national prospective cohort study. Lancet Infect Dis. 2017;17:510–9. 28139432 10.1016/S1473-3099(16)30521-7

[16] Brouwer MC, van de Beek D. Adjunctive dexamethasone treatment in adults with *Listeria monocytogenes* meningitis: a prospective nationwide cohort study. EClinicalMedicine. 2023;58: 101922. 37007737 10.1016/j.eclinm.2023.101922 PMC10050789

[17] Nau R, Wellmer A, Soto A, Koch K, Schneider O, Schmidt H, Gerber J, Michel U, Brück W. Rifampin reduces early mortality in experimental *Streptococcus pneumoniae* meningitis. J Infect Dis. 1999;179(6):1557–60. 10.1086/314760. 10228082 10.1086/314760

[18] Gerber J, Pohl K, Sander V, Bunkowski S, Nau R. Rifampin followed by ceftriaxone for experimental meningitis decreases lipoteichoic acid concentrations in cerebrospinal fluid and reduces neuronal damage in comparison to ceftriaxone alone. Antimicrob Agents Chemother. 2003;47:1313–7. 10.1128/AAC.47.4.1313-1317.2003. 12654664 10.1128/AAC.47.4.1313-1317.2003 PMC152510

[19] Grandgirard D, Burri M, Agyeman P, Leib SL. Adjunctive daptomycin attenuates brain damage and hearing loss more efficiently than rifampin in infant rat pneumococcal meningitis. Antimicrob Agents Chemother. 2012;56:4289–95. 10.1128/AAC.00674-12. 22644021 10.1128/AAC.00674-12 PMC3421563

[20] Muri L, Grandgirard D, Buri M, Perny M, Leib SL. Combined effect of non-bacteriolytic antibiotic and inhibition of matrix metalloproteinases prevents brain injury and preserves learning, memory and hearing function in experimental paediatric pneumococcal meningitis. J Neuroinflammation. 2018;15(1): 233. 10.1186/s12974-018-1272-8. 30131074 10.1186/s12974-018-1272-8 PMC6103863

